# An E xpeditious Iodine-Catalyzed Synthesis of 3-Pyrrole-substituted 2-Azetidinones

**DOI:** 10.3390/molecules171011570

**Published:** 2012-09-28

**Authors:** Debasish Bandyopadhyay, Jessica Cruz, Ram N. Yadav, Bimal K. Banik

**Affiliations:** Department of Chemistry, The University of Texas-Pan American, 1201 West University Drive, Edinburg, TX 78539, USA

**Keywords:** iodine, microwave, 2-azetidinone, pyrrole, catalysis, green synthesis

## Abstract

2-Azetidinones and pyrroles are two highly important classes of molecules in organic and medicinal chemistry. A green and practical method for the synthesis of 3-pyrrole-substituted 2-azetidinones using catalytic amounts of molecular iodine under microwave irradiation has been developed. Following this method, a series of 3-pyrrole- substituted 2-azetidinones have been synthesized with a variety of substituents at N-1 and at C-4. The procedure is equally effective for mono- as well as polyaromatic groups at the N-1 position of the 2-azetidinone ring. The C-4 substituent has no influence either on the yield or the rate of the reaction. Optically pure 3-pyrrole-substituted 2-azetidinones have also been synthesized following this methodology. No deprotection/rearrangement has been identified in this process, even with highly acid sensitive group-containing substrates. A plausible mechanistic pathway has also been suggested based on the evidence obtained from ^1^H-NMR spectroscopy. The extreme rapidity with excellent reaction yields is believed to be the result of a synergistic effect of the Lewis acid catalyst (molecular iodine) and microwave irradiation.

## 1. Introduction

Since Fleming’s discovery of penicillin from the mould *Penicillium notatum* in 1928, β-lactam antibiotics remain as one of the most important contributions of science to mankind. The 2-azetidinone (commonly known as β-lactam) ring system is the basic structural feature of a number of broad spectrum β-lactam antibiotics, including penicillins, cephalosporins, carbapenems, nocardicins, monobactams, clavulanic acid, sulbactams and tazobactams, which have been widely used as therapeutic agents to treat bacterial infections and several other diseases [1,2,3,4,5,6,7,8,9,10,11,12,13,14]. The discovery of the nocardicins and monobactams has demonstrated for the first time that β-lactams do not require a conformationally constrained bicyclic structure to possess pharmacological activities, suggesting that the medicinal activity is strictly correlated to the presence of a suitably functionalized 2-azetidinone ring. The biological activity of the β-lactams is generally believed to be associated with the chemical reactivity of the four-membered ring and on the substituents especially at nitrogen of the 2-azetidinone ring [15]. Staudinger’s ketene-imine [2+2] cycloaddition reaction [16] is the most common method for the synthesis of β-lactams. 

Like 2-azetidinones, pyrroles have occupied a central position in drug discovery since its inception (1884) over one hundred years ago, initially by reason of their occurrence in the key molecules of life, such as haemoglobin, chlorophyll, vitamin B12, porphyrins, bile pigments, coenzymes and more recently, because of their role as components of pharmaceuticals [currently, one of the best selling drugs, Lipitor (atorvastatin), has a pyrrole in its core] [17]. Pyrrole derivatives have also been reported as antimicrobial and antioxidant [18,19], anti-HIV [20], anticancer [21,22], antagonists of 5-HT7 receptor [23], antihepatitis [24] and antifungal agents [25] as well as cognition enhancers [26]. Besides these, this moiety is also present in several synthetic pharmaceuticals as well as electrically conducting polymers [27,28,29]. This general importance of pyrroles has prompted chemists to find new ways to synthesize pyrrole and its derivatives. 

**Scheme 1:**
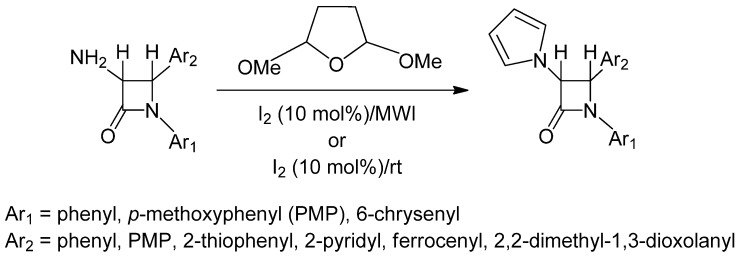
Molecular iodine-catalyzed synthesis of 3-pyrrole substituted 2-azetidinones under solventless condition.

In recent years, renewed interest has been focused on the synthesis and modiﬁcation of β-lactam ring to obtain compounds with diverse pharmacological activities. As a part of our ongoing research on synthesis of anticancer agents [30,31,32,33], some of our [34,35,36] *trans*-acetoxy β-lactams have demonstrated selective anticancer activity against a number of human cancer cell lines *in vitro* and *in vivo*. This finding has provided justification to develop an efficient synthesis of pyrrole-substituted β-lactams, based on the chemistry shown in Scheme 1.

## 2. Results and Discussion

### 2.1. Results

Molecular iodine has acquired an important role in organic synthesis due to its unique and powerful features [37,38,39,40,41,42,43,44,45] as a Lewis acid. In recent years, the use of molecular iodine in organic chemistry has received considerable attention mainly because molecular iodine is moisture-stable, less toxic than alternative acidic catalysts, inexpensive and readily available. We have demonstrated the catalytic activity of molecular iodine in several of our earlier studies which include glycosylation [46,47], Michael reaction [48], deprotection of acetals and ketals [49], synthesis of pyrroles [50] and synthesis of quinoxalines [51]. On the other hand, we have been engaged in the study of microwave-induced reactions for many years. Using microwave irradiation technique we have successfully developed several new methodologies which include stereoselective synthesis of β-lactams [52,53,54], synthesis of pyrroles [55,56,57,58], aza-Michael addition [59], hydrolysis of amides [60], and electrophilic substitution of indoles [61,62]*.* During the course of present study, it has been conceived that 3-pyrrole-substituted 2-azetidinones could easily be prepared from the corresponding primary amine using molecular iodine as the catalyst under microwave irradiation. This idea has been extended in this paper through the reaction between 3-amino-2-azetidinones with 2,5-dimethoxytetrahydrofuran in the presence of catalytic amount of molecular iodine under solvent-free condition. 

Our initial work started with screening of catalyst loading and solvent so as to identify optimal reaction conditions for the synthesis of 3-pyrrole-substituted 2-azetidinones. First of all, a number of solvents with different polarity have been screened using 1 mmol of (±)-*trans* 3-amino-1-(chrysen-6-yl)-4-phenylazetidin-2-one with 1.2 mmol of 2,5-dimethoxytetrahydrofuran using molecular iodine as catalyst (20 mol%) as a model reaction under automated CEM microwave irradiation conditions (300 Watts, 90 °C, 3 min). The results are summarized in Table 1.

**Table 1:** Microwave-assisted (300 Watts, 90 °C, 24–50 psi) synthesis of 3-pyrrole substituted 2-azetidinones from 1 mmol of (±)-*trans* 3-amino-1-(chrysen-6-yl)-4-phenylazetidin-2-one with 1.2 mmol of 2,5-dimethoxytetrahydrofuran using molecular iodine as catalyst (20 mol%) for 3 min: solvent optimization.

Entry	Solvent (1 mL)	Yield (%) ^a^
1	Water	83
2	THF	77
3	Ethanol	70
4	Toluene	51
5	Methanol	69
6	Dichloromethane	48
7	DMSO	66
**8**	**Neat**	**91**

^a^ isolated yield.

The results suggest that the absence of solvent is convenient for this reaction (Entry 8, Table 1). In the absence of any catalyst (only microwave irradiation under solventless condition) the same reaction has produced 21% yield in three minutes. The yield of the desired 2-azetidinone could be increased to 32% if the reaction is conducted for 30 minutes. The same reaction is used to optimize the amount of the catalyst to identify the best conditions (Table 2). The results show that 10 mol% molecular iodine is required to complete the reaction within three minutes (Entry 5, Table 2). 

**Table 2:** Microwave-assisted (300 Watts, 90 °C, 24–50 psi) synthesis of 3-pyrrole substituted 2-azetidinones from 1 mmol of (±)-*trans* 3-amino-1-(chrysen-6-yl)-4-phenylazetidin-2-one with 1.2 mmol of 2,5-dimethoxytetrahydrofuran using molecular iodine as catalyst under neat condition for 3 min: optimization of the amount of the catalyst.

Entry	Molecular I_2_ (mol%)	Yield (%) ^a^
1	30	78
2	25	82
3	20	91
4	15	92
**5**	**10**	**98**
6	5	69
7	2	54
8	1	47

^a^ isolated yield.

**Scheme 2:**
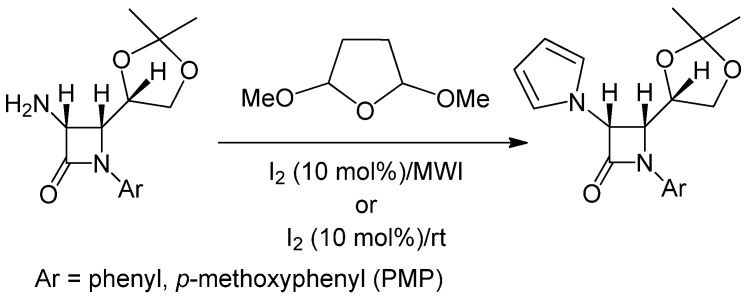
Synthesis of 3-pyrrole substituted optically pure 2-azetidinones.

Considering these observations, next we have carried out a series of reactions using various 3-amino- substituted 2-azetidinones (1 mmol), 2,5-dimethoxytetrhydrofuran (1.2 mmol) under solventless conditions under the catalytic influence of molecular iodine (10 mol%) following an automated microwave-assisted procedure (300 Watts, 90 °C, 24–50 psi; Condition A) as well as stirring the reaction mixture at room temperature (Condition B). To verify the efficacy of the newly developed method, optically pure 3-amino-2-azetidinones were subjected to the reaction conditions and the corresponding pyrrole derivatives are isolated in good to excellent yields (Entries 10, 11, Table 3), both at room temperature, as well as in the microwave-induced procedure (Scheme 2).

It is important to note that no deprotection/rearrangement is observed in the synthesis of pyrroles. The whole series of reactions was repeated at room temperature (stirring) under solventless conditions but the reactions have produced lower yields after much longer times (Table 3). The reaction has proceeded equally well irrespective of the nature of the substituents at C–4 (aryl, heteroaryl, ferrocenyl or 2,2-dimethyl-1,3-dioxolanyl). No stereochemical change has been noticed in any examples. The results obtained from both the methods (Condition A and Condition B) are summarized in Table 3. 

**Table 3:** Molecular iodine-catalyzed microwave-assisted synthesis of 3-pyrrole substituted 2-azetidinones following Scheme 1 and Scheme 2.

Entry	Substrate	Product	Condition A (MWI)		Condition B	
			300 Watts/90 °C/24–50 psi		Stirring at room	
					temperature	
			Time (min)	Yield (%) ^a^	Time (h)	Yield (%) ^a^
1	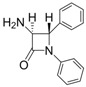	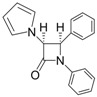	1	96	12	72
2	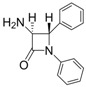	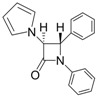	1	93	12	74
3	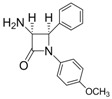	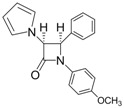	1	99	12	75
4	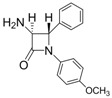	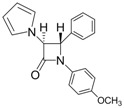	1	94	12	73
5	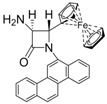	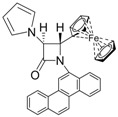	3	87	24	63
6	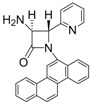	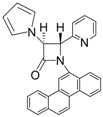	3	90	24	59
7	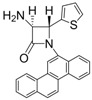	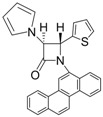	3	92	24	61
8	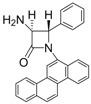	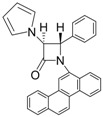	3	98	24	71
9	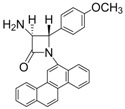	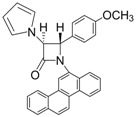	3	95	24	67
10	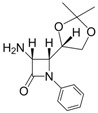	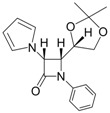	1	93	12	70
11	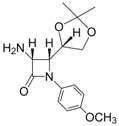	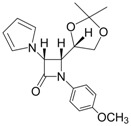	1	97	12	73

^a^ isolated yield.

It is very convenient to conduct reactions with molecular iodine because of its stability in the presence of moisture and oxygen. In all the cases, the reactions are completed within 1–3 min and the products are obtained in excellent yield (Table 3). The 3-amino-2-azetidinones were synthesized following the procedure published from our laboratory [63,64]. 

### 2.2. Discussion

**Scheme 3:**
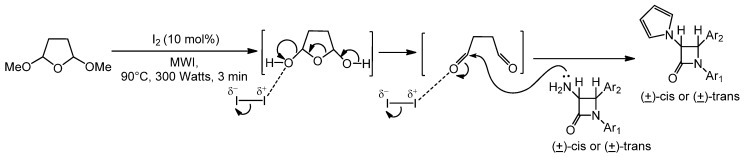
Plausible mechanistic pathway for the synthesis of 3-amino-2-azetidinones.

Reaction of *N*-aryl imines with *N*-phthaloylglycine in the presence of triethylamine and 2-chloro-*N*-methylpyridinium iodide was performed at 0 °C–room temperature. This reaction produced a mixture of (±)-*cis* and (±)-*trans* 2-azetidinones. However, polyaromatic imine derived from 6-chrysenyl amine produced exclusively (±)-*trans* 2-azetidinones. The formation of a diastereoisomeric mixture in the case of *N*-monoaromatic imines had cast doubt about the analysis of the product distributions of earlier studies [54,65,66,67], where the formation of *trans* isomer was reported. It appears that the electron- withdrawing aromatic groups at the *C*- and *N*-terminus of the imine are responsible for the formation of the *trans* isomer, whose formation was rationalized through an isomerization of the enolate as described [30,31,32,52] earlier. Polyaromatic groups (in this example, 6-chrysenyl) at the nitrogen stabilize the iminium ion greatly. The electron-withdrawing properties of monocyclic aromatic groups at the *N*-site of the imine were not sufficient to have a complete isomerization of the enolate and therefore, a mixture of *cis*- and *trans*-isomer was formed. A series of 3-pyrrole-substituted 2-azetidinones were synthesized from 3-amino-2-azetidinones in the presence of molecular iodine (10 mol%) as catalyst under microwave irradiation under solventless conditions. Although the mechanism of the reaction was not studied in detail, based on ^1^H-NMR data, a plausible mechanistic pathway may be suggested (Scheme 3).

A deprotection reaction of the methoxy groups in 2,5-dimethoxytetrahydrofuran takes place under mild acidic conditions and this process can be extremely accelerated by microwave irradiation. The intermediate dialdehyde (Scheme 3) reacts with 3-amino-β-lactams to produce the corresponding 3-pyrrole-substituted β-lactams following a nucleophilic addition and subsequent dehydration-aromatization route. This reaction suggests the competence of molecular iodine to serve as a Lewis activator. ^1^H-NMR spectroscopy has been used to provide additional support for the proposed mechanism. Upon irradiating a CDCl_3_ solution of 2,5-dimethoxytetrahydrofuran for 3 min in the presence of a catalytic amount of iodine, a ^1^H-NMR was taken. A highly intense downfield signal due to the –CHO group is observed. This clearly suggests the formation of the reactive dialdehyde in the reaction media. Under the influence of the electrophilic reagent, molecular iodine, demethylation of the methoxy groups in 2,5-dimethoxytetrahydrofuran takes place. 

Application of microwave technology to the rapid synthesis of biologically significant heterocyclic molecules under solvent-free condition is very promising and challenging. The ultimate aim, of course is to conduct the reactions under solvent-free condition [68]. Development of cleaner technologies is a major emphasis in green chemistry. The combination of solvent-free reaction condition and microwave-irradiation is used as an eco-friendly approach for the synthesis of a variety of products and this generally leads to reduction in reaction times, enhances conversions, and changes of stereoselectivity of the product. Microwaves, as a part of the electromagnetic spectrum, are composed of two field components: electric and the magnetic. For the purpose of heating, the electric component is important as it results in a force being applied to all the polar or charged species. Such species, in response to the electric field, start to move or rotate and this causes additional polarization of the polar species in the vicinity. When dipolar species are subjected to the electric component of microwave fields they start to oscillate, following the oscillation of the electric field. During such oscillation, the polar or charged species collide with neighboring particles (charged or neutral). This rapid motion and resulting intermolecular friction cause an intense internal heat that can increase the rate of reaction [69,70]. It is obvious that the dielectric properties of the material under consideration are of paramount importance. In presence of microwaves, molecular iodine increases the “anionic activation” [71] because of its Lewis acidic character. Due to this reason the relative permittivity (ability of a molecule to be polarized by the application of an electric field) of the carbonyl groups, of the intermediate dialdehyde increases (Scheme 3), which facilitates microwave heating extensively. The larger the relative permittivity of a substance, the greater will be the coupling with microwaves [72]. When the reagents are subjected to microwave irradiation, microwaves pass through the (glass) walls of the reaction vessel and only heat the reactants, avoiding local overheating at the reaction walls. This can eliminate formation of side products and helps to explain the higher yields and purities typically observed. The extreme rapidity with excellent yield of the reaction can be rationalized as a synergistic effect of the Lewis acid catalyst (molecular iodine) and microwave irradiation.

## 3. Experimental

### 3.1. General

Melting points were determined in a Fisher Scientific electrochemical Mel-Temp* manual melting point apparatus (Model 1001) equipped with a 300 °C thermometer. FT-IR spectra were registered on a Bruker IFS 55 Equinox FTIR spectrophotometer as KBr discs. ^1^H-NMR (600 MHz) and ^13^C-NMR (150 MHz) spectra were obtained at room temperature with Bruker superconducting Ultrashield^TM^ Plus 600 MHz NMR spectrometer with central field 14.09 Tesla, coil inductance 89.1 Henry and magnetic energy 1127.2 kJ using CDCl_3_ as solvent. Elemental analyses (C, H, N) were conducted using the Perkin-Elmer 2400 series II elemental analyzer, their results were found to be in good agreement (±0.2%) with the calculated values for C, H, N. All the reagents (analytical grade) were purchased from Sigma-Aldrich Corporation (St. Louis, MO, USA). Throughout the project solvents were purchased from Fisher-Scientific (Pittsburgh, PA, USA). Deionized water was used for the preparation of all aqueous solutions. 

### 3.2. General Procedure for the Synthesis of 3-Pyrrole Substituted 2-Azetidinones from 3-Amino-2-azetidinones under Microwave Irradiation

The substrate (3-amino-2-azetidinones, 1.0 mmol), 2,5-dimethoxytetrahydrofuran (1.2 mmol) and iodine (10 mol%) were mixed together in a microwave vial. A small magnetic stir bar was placed in the reaction mixture. The mixture was irradiated in an automated microwave (CEM Corporation, Matthews, NC, USA) and the progress of the reaction was monitored by TLC. After completion of the reaction (Table 3), diethyl ether (10 mL) was added to the reaction mixture and the organic layer was washed with saturated sodium bicarbonate solution, brine and water successively. It was dried over anhydrous sodium sulfate and the solvent was removed under reduced pressure. The crude mass was purified through a small silica gel column using ethyl acetate/hexanes as eluent. The physical and spectral data [73] of the 3-pyrrole substituted 2-azetidinones are as follows:

*(*±*)-cis-1,4-Diphenyl-3-(1H-pyrrol-1-yl)azetidin-2-one (Entry 1, Table 3).* White solid (96%); mp 158 °C; IR: 3247, 2913, 1759, 1516, 1450, 1291, 1115, 712 cm^−1^; ^1^H-NMR: δ 5.43 (d, *J* = 5.41 Hz, 1H), 5.74 (d, *J* = 5.40 Hz, 1H), 5.87 (t, *J* = 2.04 Hz, 2H), 6.47 (t, *J* = 2.04, 2H), 7.14–7.42 (m, 10H); ^13^C-NMR: δ 62.12, 68.43, 109.08, 117.90, 120.44, 125.05, 126.77, 128.60, 129.59, 132.67, 137.84, 162.03. Anal. Calcd for C_19_H_17_N_2_O: C, 79.14; H, 5.59; N, 9.72. Found: C, 79.01; H, 5.52; N, 9.63.

*(*±*)-trans-1,4-Diphenyl-3-(1H-pyrrol-1-yl)azetidin-2-one (Entry 2, Table 3).* White solid (93%); mp 155 °C; IR: 3128, 2945, 2914, 1750, 1593, 1493, 1447, 1375, 1317, 1222, 1133, 1084, 961, 896, 728, 705 cm^−1^; ^1^H-NMR: δ 4.97 (d, *J* = 1.92 Hz, 1H), 5.12 (d, *J* = 2.04 Hz, 1H), 6.26 (t, *J* = 1.98 Hz, 2H), 6.77 (t, *J* = 1.92 Hz, 2H), 7.09–7.42 (m, 10H); ^13^C-NMR: δ 65.36, 73.25, 110.08, 117.68, 119.58, 124.78, 125.89, 126.01, 129.14, 129.48, 135.68, 136.78, 161.93. Anal. Calcd for C_19_H_17_N_2_O: C, 79.14; H, 5.59; N, 9.72. Found: C, 79.08; H, 5.56; N, 9.65.

*(*±*)-cis-1-(4-Methoxyphenyl)-4-phenyl-3-(1H-pyrrol-1-yl)azetidin-2-one (Entry 3, Table 3).* White crystalline solid (99%); mp 162 °C; IR: 3060, 2966, 1742, 1510, 1488, 1388, 1297, 1242, 1172, 1092, 808, 725, 692 cm^−1^; ^1^H-NMR: δ 3.77 (s, 3H), 5.39 (d, *J* = 5.28 Hz, 1H), 5.76 (d, *J* = 5.34 Hz, 1H), 5.88 (t, *J* = 2.04 Hz, 2H), 6.47 (t, *J* = 1.98 Hz, 2H), 6.83 (d, *J* = 9.0 Hz, 2H), 7.11–7.27 (m, 5H), 7.36 (d, *J* = 9.0 Hz, 2H); ^13^C-NMR: δ 55.48, 62.02, 68.23, 109.04, 114.48, 118.79, 120.17, 126.65, 128.31, 128.53, 130.69, 132.55, 156.66, 161.07. Anal. Calcd for C_20_H_18_N_2_O_2_: C, 75.45; H, 5.70; N, 8.80. Found: C, 75.31; H, 5.63; N, 8.72. 

*(*±*)-trans-1-(4-Methoxyphenyl)-4-phenyl-3-(1H-pyrrol-1-yl)azetidin-2-one (Entry 4, Table 3).* White crystalline solid (94%); mp 145 °C; IR: 3126, 2958, 2931, 1757, 1728, 1514, 1463, 1450, 1382, 1321, 1288, 1258, 1135, 1091, 1069, 1036, 827, 740, 727 cm^−1^; ^1^H-NMR: δ 3.74 (s, 3H), 4.93 (d, *J* = 2.04 Hz, 1H), 5.10 (d, *J* = 2.04 Hz, 1H), 6.25 (t, *J* = 2.16 Hz, 2H), 6.76 (t, *J* = 2.10 Hz, 2H), 6.80 (d, *J* = 2.16 Hz, 1H), 6.81 (d, *J* = 2.22 Hz, 1H), 7.28–7.40 (m, 7H); ^13^C-NMR: δ 55.45, 65.44, 73.24, 110.00, 114.47, 119.02, 119.56, 125.95, 129.10, 129.44, 130.87, 135.74, 156.65, 161.29. Anal. Calcd for C_20_H_18_N_2_O_2_: C, 75.45; H, 5.70; N, 8.80. Found: C, 75.36; H, 5.59; N, 8.74. 

*(*±*)-trans-1-(Chrysen-6-yl)-4-(ferrocenyl)-3-(1H-pyrrol-1-yl)azetidin-2-one (Entry 5, Table 3).* Orange crystalline solid (87%); mp 208 °C; IR: 2360, 1754, 1593, 1490, 1439, 1381, 1314, 1105, 819, 756, 727 cm^−1^; ^1^H-NMR: δ 3.82 (s, 5H), 4.02 (m, 1H), 4.07 (m, 1H), 4.16 (m, 1H), 4.24 (m, 1H), 5.29 (d, *J* = 2.28 Hz, 1H), 5.43 (d, *J* = 2.28 Hz, 1H), 6.32 (t, *J* = 2.10 Hz, 2H), 6.98 (d, *J* = 2.28 Hz, 2H), 7.48–8.76 (m, 11H); ^13^C-NMR: δ 65.72, 68.58, 68.68, 69.03, 69.43, 70.84, 82.63, 110.28, 117.96, 119.83, 120.92, 123.00, 123.81, 123.93, 126.82, 127.01, 127.10, 127.47, 127.82, 127.99, 128.13, 128.69, 130.15, 130.64, 131.54, 132.28, 164.27. Anal. Calcd for C_35_H_26_FeN_2_O: C, 76.93; H, 4.80; N, 5.13. Found: C, 76.81; H, 4.69; N, 5.06. 

*trans-1-(Chrysen-6-yl)-4-(pyridin-2-yl)-3-(1H-pyrrol-1-yl)azetidin-2-one (Entry 6, Table 3).* White solid (90%); mp 224 °C; IR: 3057, 1756, 1591, 1488, 1471, 1437, 1393, 1318, 1141, 1095, 1070, 816, 761 cm^−1^; ^1^H-NMR: δ 5.56 (d, *J* = 1.68 Hz, 1H), 5.88 (d, *J* = 1.68 Hz, 1H), 6.35 (t, *J* = 1.92 Hz, 2H), 7.02 (t, *J* = 1.95 Hz, 2H), 7.16–8.78 (m, 15H); ^13^C-NMR: δ 68.18, 70.19, 110.10, 117.45, 119.98, 120.82, 122.63, 123.91, 124.10, 126.96, 127.03, 127.45, 127.51, 127.88, 128.02, 128.62, 130.10, 130.76, 131.49, 132.18, 136. 94, 150.54, 154.62, 163.99. Anal. Calcd for C_30_H_21_N_3_O: C, 81.98; H, 4.82; N, 9.56. Found: C, 81.89; H, 4.76; N, 9.49.

*(*±*)-trans-1-(Chrysen-6-yl)-3-(1H-pyrrol-1-yl)-4-(thiophen-2-yl)azetidin-2-one (Entry 7, Table 3).* Yellow solid (92%); mp 150 °C; IR: 1762, 1593, 1487, 1438, 1389, 1371, 816 cm^−1^; ^1^H-NMR: δ 5.58 (d, *J* = 2.04 Hz, 1H), 5.76 (d, *J* = 1.98 Hz, 1H), 6.36 (t, *J* = 1.92 Hz, 2H), 6.88 (dd, *J* = 3.78 Hz, 1.08 Hz, 1H), 6.98 (t, *J* = 2.04 Hz, 2H), 7.11 (d, *J* = 3.36 Hz, 1H), 7.23 (d, *J* = 4.98 Hz, 1H), 7.61–8.79 (m, 11H); ^13^C-NMR: δ 63.87, 72.90, 110.28, 110.36, 116.99, 119.74, 119.83, 120.85, 122.91, 123.73, 124.02, 126.56, 126.81, 126.99, 127.08, 127.29, 127.32, 127.42, 127.50, 128.01, 128.12, 128.66, 130.08, 130.19, 131.56, 132.22, 138.95, 163.22. Anal. Calcd for C_29_H_20_N_2_OS: C, 78.35; H, 4.53; N, 6.30. Found: C, 78.21; H, 4.45; N, 6.24.

*(*±*)-trans-1-(Chrysen-6-yl)-4-phenyl-3-(1H-pyrrol-1-yl)azetidin-2-one (Entry 8, Table 3).* White solid (98%); mp 124 °C; IR: 2919, 2353, 2323, 1762, 1707, 1593, 1488, 1455, 1438, 1387, 1346, 1314, 1092, 1070, 817 cm^−1^; ^1^H-NMR: δ 5.44 (d, *J* = 2.02 Hz, 1H), 5.54 (d, *J* = 2.02 Hz, 1H), 6.35 (t, *J* = 2.08 Hz, 2H), 6.96 (t, *J* = 2.12 Hz, 2H), 7.27–7.98 (m, 11H), 8.41–8.81 (m, 11H); ^13^C-NMR: δ 67.51, 7191, 110.25, 115.53, 119.74, 120.87, 122.85, 123.72, 124.30, 126.33, 126.79, 126.92, 127.03, 127.46, 127.50, 127.72, 127.92, 128.67, 129.21, 129.35, 130.01, 130.89, 131.61, 132.24, 135.71, 163.46. Anal. Calcd for C_31_H_22_N_2_O: C, 84.91; H, 5.06; N, 6.39. Found: C, 84.77; H, 4.97; N, 6.31.

*(*±*)-trans-1-(Chrysen-6-yl)-4-(4-methoxyphenyl)-3-(1H-pyrrol-1-yl)azetidin-2-one (Entry 9, Table 3).* Light yellow solid (95%); mp 160 °C; IR: 3099, 2990, 2357, 1755, 1603, 1509, 1478, 1439, 1232, 1173, 1142, 1095, 1017, 958, 821, 743 cm^−1^; ^1^H-NMR: δ 3.71 (s, 3H), 5.35 (d, *J* = 2.22 Hz, 1H), 5.41 (d, *J* = 2.16 Hz, 1H), 6.27 (t, *J* = 2.10 Hz, 2H), 6.74 (d, *J* = 8.64 Hz, 2H), 6.88 (t, *J* = 2.10 Hz, 2H), 7.31 (d, *J* = 8.58 Hz, 2H), 7.63–8.82 (m, 11H); ^13^C-NMR: δ 55.27, 67.35, 71.99, 110.17, 114.75, 115.77, 119.74, 120.87, 122.88, 123.71, 124.27, 126.77, 126.91, 127.00, 127.01, 127.45, 127.48, 127.69, 127.77, 127.88, 128.66, 130.03, 130.81, 131.59, 132.24, 160.28, 163.63. Anal. Calcd for C_32_H_24_N_2_O_2_: C, 82.03; H, 5.16; N, 5.98. Found: C, 81.96; H, 5.11; N, 5.90.

*(3R,4R)-4-((S)-2,2-Dimethyl-1,3-dioxolan-4-yl)-1-phenyl-3-(1H-pyrrol-1-yl)azetidin-2-one (Entry 10, Table 3).* Pale yellow crystalline solid (93%); mp 131 °C; IR: 2918, 1766, 1593, 1570, 1370, 1204, 1095, 822, 730, 691 cm^−1^; ^1^H-NMR: δ 1.17 (s, 3H), 1.40 (s, 3H), 2.88 (t, *J* = 7.02 Hz, 1H), 3.26 (m, 1H), 4.05 (dd, *J* = 11.01, 8.16 Hz, 1H), 4.29 (m, 1H), 5.44 (d, *J* = 5.58 Hz, 1H), 6.16 (s, 2H), 6.62 (s, 2H), 7.09 (t, *J* = 7.38 Hz, 1H), 7.29 (t, *J* = 7.80 Hz, 2H), 7.73 (d, *J* = 8.22 Hz, 2H); ^13^C-NMR: δ 25.12, 26.52, 62.91, 64.82, 65.76, 77.21, 109.81, 110.55, 118.78, 120.69, 124.99, 128.98, 137.55, 162.19. Anal. Calcd for C_18_H_20_N_2_O_3_: C, 69.21; H, 6.45; N, 8.97. Found: C, 69.11; H, 6.40; N, 8.90.

*(3R,4R)-4-((S)-2,2-Dimethyl-1,3-dioxolan-4-yl)-1-(4-methoxyphenyl)-3-(1H-pyrrol-1-yl)azetidin-2-one (Entry 11, Table 3).* White crystalline solid (97%); mp 142 °C; IR: 3122, 2986, 1738, 1514, 1385, 1237, 832, 736 cm^−1^; ^1^H-NMR: δ 1.18 (s, 3H), 1.39 (s, 3H), 2.87 (dd, *J* = 7.74, 3.24 Hz, 1H), 3.26 (t, *J* = 8.22 Hz, 1H), 3.74 (s, 3H), 4.03 (dd, *J* = 11.37, 7.32 Hz, 1H), 4.23 (dd, *J* = 7.08, 3.12 Hz, 1H), 5.41 (d, *J* = 5.52 Hz, 1H), 6.16 (s, 2H), 6.61 (s, 2H), 6.82 (d, *J* = 8.88 Hz, 2H), 7.67 (d, *J* = 8.88 Hz, 2H); ^13^C-NMR: δ 25.15, 26.54, 55.50, 63.04, 64.85, 65.78, 77.20, 109.78, 110.49, 114.09, 119.81, 120.68, 131.02, 156.84, 161.61. Anal. Calcd for C_19_H_22_N_2_O_4_: C, 66.65; H, 6.48; N, 8.18. Found: C, 66.49; H, 6.36; N, 8.04.

## 4. Conclusions

In summary, an extremely rapid, convenient and environmentally benign route for the one-step synthesis of 3-pyrrole-substituted 2-azetidinones was developed. The present simple methodology offers attractive features such as short reaction times and milder conditions, and produces the products in excellent yields. The procedure is equally effective with mono- and polyaromatic 2-azetidinones. This strategy should find application in the synthesis of various organic compounds of medicinal interest. 
